# Reference Genes in the Pathosystem *Phakopsora pachyrhizi*/ Soybean Suitable for Normalization in Transcript Profiling

**DOI:** 10.3390/ijms160923057

**Published:** 2015-09-23

**Authors:** Daniela Hirschburger, Manuel Müller, Ralf T. Voegele, Tobias Link

**Affiliations:** Department of Phytopathology, Institute of Phytomedicine, Faculty of Agricultural Sciences, University of Hohenheim, Otto-Sander-Straße 5, 70599 Stuttgart, Germany; E-Mails: Daniela.Hirschburger@uni-hohenheim.de (D.H.); Manuel.Mueller@uni-hohenheim.de (M.M.); Ralf.Voegele@uni-hohenheim.de (R.T.V.)

**Keywords:** *Phakopsora pachyrhizi*, RT-qPCR, reference gene, HIGS/VIGS, effector

## Abstract

*Phakopsora pachyrhizi* is a devastating pathogen on soybean, endangering soybean production worldwide. Use of Host Induced Gene Silencing (HIGS) and the study of effector proteins could provide novel strategies for pathogen control. For both approaches quantification of transcript abundance by RT-qPCR is essential. Suitable stable reference genes for normalization are indispensable to obtain accurate RT-qPCR results. According to the *M*inimum *I*nformation for Publication of *Q*uantitative Real-Time PCR *E*xperiments (MIQE) guidelines and using algorithms geNorm and NormFinder we tested candidate reference genes from *P. pachyrhizi* and *Glycine max* for their suitability in normalization of transcript levels throughout the infection process. For *P. pachyrhizi* we recommend a combination of *CytB* and *PDK* or *GAPDH* for *in planta* experiments. Gene expression during *in vitro* stages and over the whole infection process was found to be highly unstable. Here, *RPS14* and *UbcE2* are ranked best by geNorm and NormFinder. Alternatively *CytB* that has the smallest *C*q range (*C*q: quantification cycle) could be used. We recommend specification of gene expression relative to the germ tube stage rather than to the resting urediospore stage. For studies omitting the resting spore and the appressorium stages a combination of *Elf3* and *RPS9*, or *PKD* and *GAPDH* should be used. For normalization of soybean genes during rust infection *Ukn2* and *cons7* are recommended.

## 1. Introduction

*Phakopsora pachyrhizi*, the causative agent of Asian soybean rust, is one of the most devastating pathogens of soybean. The pathogen originates from East Asia, but by way of Hawaii and Australia it spread to South America where it now also causes enormous damages, especially in Brazil. In severely infected fields the pathogen can cause yield losses of up to 80%. Since 2004 the pathogen is also present in the continental United States, the biggest soybean producer worldwide, threatening production there [1].

*P. pachyrhizi* belongs to the Pucciniales, the rust fungi. It is an obligate biotrophic pathogen, which means that it is entirely dependent on its host plant for nutrient supply. Rust fungi also only sporulate growing on their respective host plant [2]. In contrast to other rust fungi that produce up to five different spore forms, for *P. pachyrhizi* infection only from urediospores has been observed. On the host leaf the urediospore attaches to the surface, germinates, forms an appressorium, and then the fungus penetrates directly through the epidermis—a feature unique to *P. pachyrhizi*. The epidermal cell that is penetrated dies in the process [1]. Afterwards the fungus grows intercellularly; the only intracellular structures formed are haustoria, the structures responsible for nutrient uptake. Seven to nine days after infection sporulation begins. Of the whole infection process only germination and appressorium formation can be induced *in vitro*. Under laboratory conditions spores can be germinated in watery suspension or on the water surface [3]. On artificial surfaces, for example polyethylene (PE) membranes, also the formation of appressoria can be induced. Since later structures cannot be observed *in vitro* it is assumed that at this point a major metabolic switch occurs and that only after this point the biotrophic interaction with the plant begins, which is established after formation of haustoria. To identify genes involved in the biotrophic interaction it is therefore common to compare gene expression in germinated spores or *in vitro* formed appressoria with gene expression in the infected leaf or in fungal structures isolated from infected leaves, for example haustoria [4,5,6]. An important practical reason to use the *in vitro* approach is that very early in the infection process the fungal biomass on an inoculated leaf is so small [7], that fungal gene expression cannot be properly quantified.

To elucidate the biotrophic interaction in more detail, genes responsible for nutrient uptake and involved in primary metabolism have been investigated. Results from these experiments support the idea that a major switch in the fungal metabolism occurs after the plant is penetrated [8,9]. A current focus in research of plant pathogens is on the so-called ‘effectors’, or their action. Effectors are defined as proteins secreted by the pathogen and transferred to the host plant to influence host metabolism. Functions of effectors can be suppression of host defense responses or to induce the release of nutrients by the host. If the host is able to recognize such effector proteins by special receptors—resistance proteins—effectors can also have the unintended effect of inducing resistance. In this case they are called avirulence proteins [10,11]. An important part in characterizing effector genes is to study their expression during the infection process.

Since rust fungi cannot be cultivated *in vitro*, it is almost impossible to genetically modify them. The system for transforming *Melampsora lini* that depends on selection for host resistance [12] so far is the exception to the rule. In consequence, other ways to influence the pathogen have come to the fore. One way to influence gene expression of plant pathogens is Host Induced Gene Silencing (HIGS). Here, the host plant is made to produce double stranded RNA (dsRNA), complementary to a pathogen gene (the messenger RNA sequence). Either the dsRNA or the downstream siRNA (small interfering RNA) that results from cleavage of the dsRNA by the RNA interference (RNAi) machinery of the host plant, notably the Dicer and Argonaute protein complexes, is transferred to the pathogen and there leads to silencing of the targeted gene. Apart from producing transgenic plants carrying constructs to produce inhibitory RNA, plant viruses can be used to introduce dsRNA [13] into the host plant. Thus, Virus Induced Gene Silencing (VIGS) has become a common tool in plant research. Since in this special case VIGS is used to perform HIGS, we would suggest the term HIGS by VIGS (HbV).

Transcript profiling to measure gene expression is an important part of characterizing pathogen genes, effectors as well as genes involved in metabolism or signaling. The effectiveness of HIGS is also tested by measuring transcript reduction of the target gene. Today the gold standard for measuring transcript levels is RT-qPCR (reverse transcript quantitative PCR).

Transcript levels are normally given relative to a starting sample and they need to be normalized by biomass, total RNA amount, or—most common, time efficient, and most exact—stably expressed genes, so-called reference genes [14]. It is common to use housekeeping genes—genes involved in primary metabolic processes—for normalization, assuming that these important genes need to be expressed unchangingly during every stage of development or in every tissue. However, this assumption is often wrong; many housekeeping genes have been proven to be regulated [15,16]. It is therefore necessary to experimentally determine stably expressed genes that can be used as reference genes. Even more so, since genes that are stable under one set of conditions can be unstable under a different set of conditions it is necessary to determine suitable reference genes for every experiment or every set of samples to be tested [14]. Since stability of expression can only be measured relative to other genes, reference genes are determined by choosing from a set of candidate reference genes comprising housekeeping genes and/or genes that were proven stable in other experiments.

Here, we determined *P. pachyrhizi* reference genes suitable for normalization in transcript profiling during the infection process including *in vitro* stages and in measuring transcript levels in HbV experiments. We also determined *Glycine max* reference genes for normalizing transcript levels of soybean genes during infection with *P. pachyrhizi*.

## 2. Results and Discussion

### 2.1. Choice of Samples

*In vitro* structures of *P. pachyrhizi* normally tested in gene expression studies are resting urediospores (s), germ tubes (gt), and appressoria (ap). Therefore, these stages were also included as samples in this study. We also included a wide range of time points of infected leaves. Non-infected leaves (ni), leaves 6 h post inoculation (hpi), 12, 18, 24, 48, 72, 120, 168, and 336 hpi. Time points 6–72 hpi cover the early stages of infection since during this time strong changes in plant gene expression have been observed [17]. Later time points (168 and 336 hpi) represent the sporulation stage and also take into account leaf senescence that is caused by strong *P. pachyrhizi* infection. Silencing efficiency in our own work using HbV is usually checked at 168 hpi. In order to ensure that our reference genes are not affected by virus infection, we included samples of plants infected with Bean Pod Mottle Virus (BPMV) (21 days post infection (dpi)) and plants infected with both BPMV and *P. pachyrhizi* (168 hpi).

All samples from leaves were used to determine *G. max* reference genes. The amount of fungal biomass and therefore the fraction of fungal RNA in infected leaves during early infection, however, is very low. As a consequence *C*q values for any fungal gene at these time points are very high and reliable determination of fungal gene expression at these time points is almost impossible. Therefore, the first of the infected leaf samples used for determining *P. pachyrhizi* reference genes was 72 hpi.

### 2.2. Choice of Candidate Reference Genes

Housekeeping genes are generally considered good candidates for reference genes. We kept to this rule and to further enhance our chances for choosing stable genes we selected genes that were identified as stable in earlier studies.

For *P. pachyrhizi* to stay close to the system, we chose from studies dealing with rust fungi. Schmitz *et al.* [18] worked with soybean rust and mentioned determining reference genes. The other studies deal with *Hemileia vastatrix* [19], *Melampsora larici-populina* [20], and *Puccinia triticina* [21]. In addition five more housekeeping genes were chosen. The actual sequences used were taken from data published by Link *et al.* [9]. The candidate reference genes are presented in Table 1.

**Table 1:** Candidate reference genes for *P. pachyrhizi*.

Gene Designation	Description	GenBank Accession/Name	Reference
*Act*	actin	GACM01001510/TSA:GACM01:Pp_contig05019	[18]
*ASUB*	ATP synthase β subunit	PpGI_Contig363rc ^a^	-
*aTub*	α tubulin	GACM01003080/TSA:GACM01:Pp_contig00787	[20]
*CytB*	cytochrome b	GQ332420	[18,19]
*Elf1a*	elongation factor 1 α	GACM01002155/TSA:GACM01:Pp_contig07365	-
*Elf3*	elongation factor 3	GACM01003209/TSA:GACM01:Pp_contig00583	-
*GAPDH*	glyceraldehyde-3-phosphate dehydrogenase	GACM01002868/TSA:GACM01:Pp_contig00008	[20]
*PDH*	pyruvate dehydrogenase	GACM01002893/TSA:GACM01:Pp_contig00239	-
*PDK*	pyruvate dehydrogenase kinase	GACM01003388/TSA:GACM01:Pp_contig02726	-
*RPS9*	40S ribosomal protein S9	GACM01002779/TSA:GACM01:Pp_contig00421	[19]
*RPS11*	40S ribosomal protein S11	GACM01003017/TSA:GACM01:Pp_contig00682	[19]
*RPS14*	40S ribosomal protein S14	GACM01002939/TSA:GACM01:Pp_contig00153	[19]
*SDH*	succinate dehydrogenase	GACM01002923/TSA:GACM01:Pp_contig00241	[21]
*Ubc*	ubiquitin	GACM01002338/TSA:GACM01:Pp_contig00942	[21]
*UbcE2*	ubiquitin conjugated enzyme	GACM01000888/TSA:GACM01:Pp_contig06751	[21]

^a^ No NCBI accession. See [9] supplementary information for sequence.

For soybean, several studies to determine reference genes have been performed [22,23,24]. One study used *G. max* homologs of housekeeping genes of *Arabidopsis thaliana* and validated and ranked them based on stable expression in different organs, under different light regimes, and in different cultivars [22]. Another study [23] searched for new reference genes, using a large dataset of different microarrays to find promising candidates and then validated and ranked these with qRT-PCR data. These authors even included soybean rust infected plant material, however just one sample. A third study [24] compared the classical housekeeping genes [22] to the new ones [23], using an even bigger set of samples. Apart from providing a good source to choose candidate genes (Table 2), these studies also found that no reference gene was stable over all sample sets analyzed and that different genes were the most stable in different sample sets. This once more highlights the necessity for validating reference genes for any given experiment.

**Table 2:** Candidate reference genes for *G. max*.

Gene Designation ^a^	Description	Gene ID	Reference
*cons7*	metalloprotease	AW310136 ^b^	[23]
*cons15*	CDPK-related protein kinase	AW396185 ^b^	[23]
*CYP2*	cyclophilin	TC224926 ^c^	[22,24]
*ELF1B*	elongation factor 1 β	TC203623 ^c^	[22,24]
*SKIP16*	F-box protein	CD397253 ^b^	[23,24]
*TIP41*	TAP42 interacting protein-signaling	EV263725 ^b^	[24]
*Ukn1*	hypothetical protein/possibly ABC transporter	BU578186 ^b^	[23,24]
*Ukn2*	hypothetical protein	BE330043 ^b^	[24]

^a^ abbreviations as used in the main text; ^b^ NCBI accession numbers; ^c^ soybase accession numbers.

### 2.3. Reference Genes for P. pachyrhizi: All Tested Genes Vary in Expression for in Vitro Structures but Are Stable in in planta Samples

Using the method described by Pfaffl [25], we first determined amplification efficiencies of the selected primers. The calculated efficiencies can be found in Table 3.

**Figure 1:**
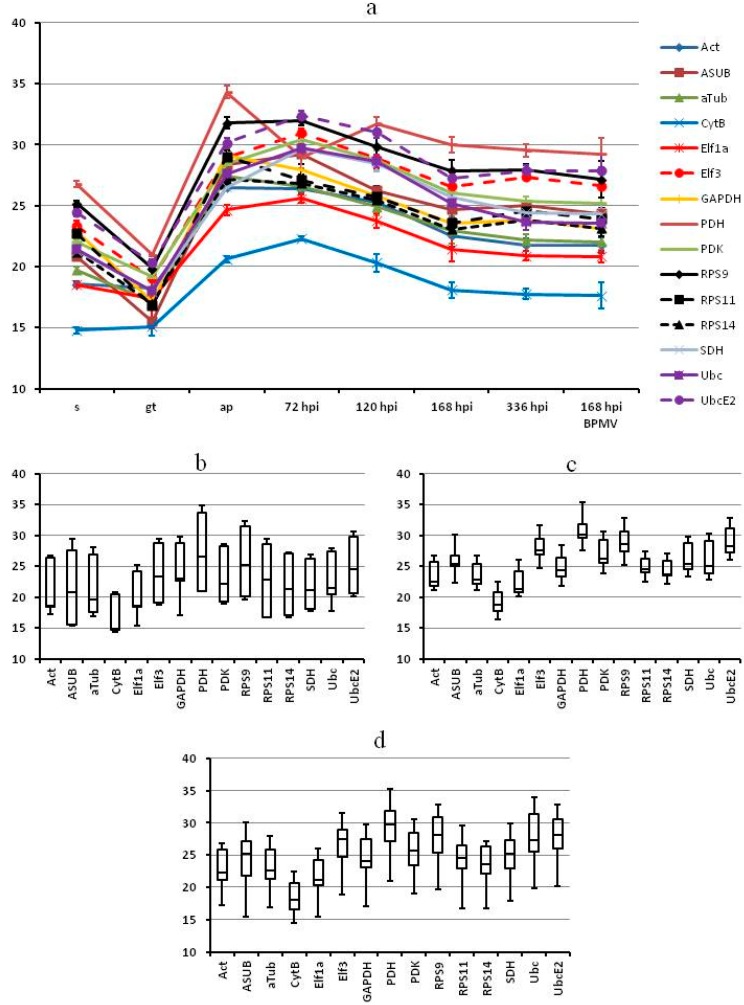
Variation of *C*q values for *P. pachyrhizi* candidate reference genes between samples (vertical axis: *C*q). (**a**) Average and standard deviation of *C*q values for every sample; (**b**–**d**) box plots representing the range of *C*q values; (**b**) *in vitro* samples; (**c**) *in planta* samples; (**d**) all samples. For better representation of RNA abundance the *C*q values were corrected for primer efficiency: *C*q_corr_ = lg_2_ ((2 × e)*^C^*^q^).

**Figure 2:**
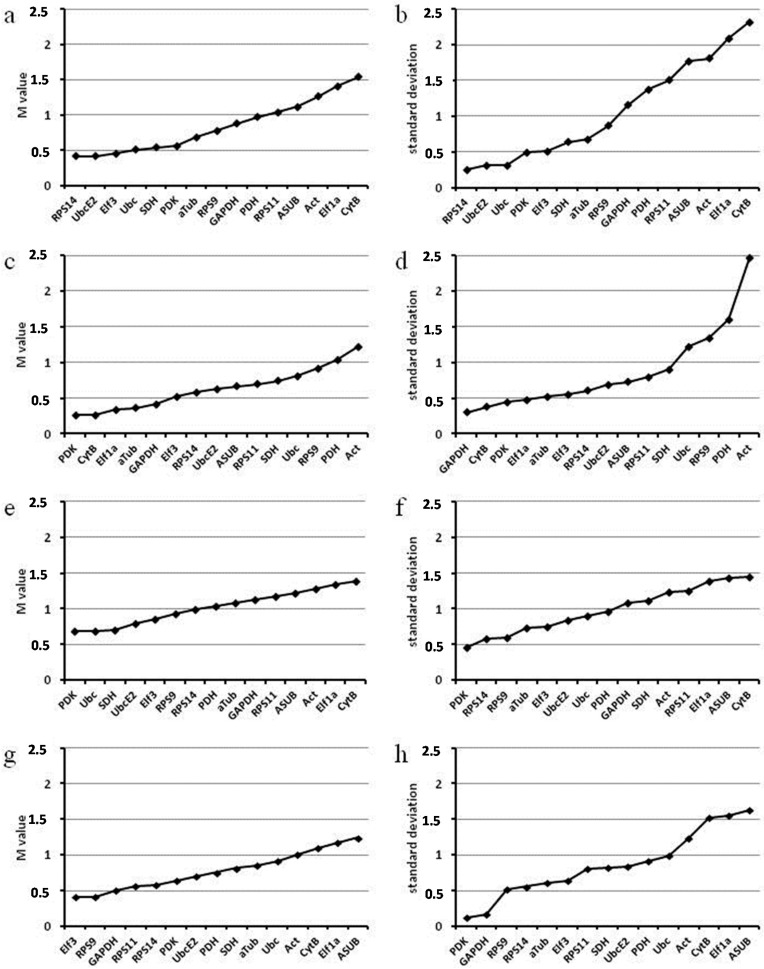
Ranking of *P. pachyrhizi* candidate reference genes for stability by the algorithms geNorm (**a**,**c**,**e**,**g**) and NormFinder (**b**,**d**,**f**,**h**). Analyses performed for (**a**,**b**) *in vitro* samples; (**c**,**d**) *in planta* samples; (**e**,**f**) all samples; (**g**,**h**) *in planta* samples together with germ tube (gt) sample; M-value: gene-stability measure calculated by geNorm—the arithmetic mean of all pairwise variations of the respective gene against all other genes between the different samples; standard deviation (here): stability measure calculated by NormFinder based on group wise comparisons.

We then performed RT-qPCR for all samples—*in vitro* structures and infected leaves starting at 72 hpi. Considering primer efficiencies we first plotted corrected *C*q values over time (Figure 1) and then used the algorithms geNorm [26] and NormFinder [27] to determine the most stable genes (Figure 2). To be able to detect differences between different stages in rust development we analyzed the samples from *in vitro* structures separately from the samples from infected leaf stages and also did an analysis that combined all samples.

In our plots of the *C*q values there is a strong variation between samples. Most genes are highly expressed in the germ tube stage and much less in the appressorium stage and in all the *in planta* stages (Figure 1a). There seems to be considerable co-regulation or rather a generally higher transcriptional activity in the germ tube than in all the other stages.

**Table 3:** Primers used in qRT-PCR for *P. pachyrhizi* genes.

Name	Gene	Sequence	Reference	Amplicon (bp)	Efficiency (%)
ActinDis 1 f (KES 1461 fw)	*Act*	acagtttcaccacaaccgcc	[18]	144	93
ActinDis 1 r (KES 1462 rv)		tgaccgtcgggaagttcg			
qPpGI363fw	*ASUB*	tgtcccaaccctttgctgtt	this study	146	86
qPpGI363rv		ggcaccgaccatgtaaaacg			
AtubDis 1 f (KES 1463 fw)	*aTub*	ctgcgaacaactatgctcgtc	[18]	116	90
AtubDis 1 r (KES 1464 fw)		cacgaagaagccttggagtcc			
CytB 1 f (KES 1688 fw)	*CytB*	tcaagacgcatccaattctaggtc	[28]	138	85
CytB 1 r (KES 1689 rv)		gtgttacacccgtgataatctgaatgat			
Elf1a 1 f	*Elf1a*	gtgagcgtggtatcaccatc	this study	143	92
Elf1a 1 r		cagaatggcgcaatcagc			
q00583fw	*Elf3*	aatgcgtggtctctctggtg	this study	89	95
q00583rv		gctcgtccaagatcaccaca			
GAPDH 1 f (KES 1465 fw)	*GAPDH*	ggtatggctttccgagttcca	[18]	157	90
GAPDH 1 r (KES 1466 rv)		tcagttgataccaaatcatcctcag			
q00239fw	*PDH*	gcggaaggaaaggataagggg	this study	136	95
q00239rv		tccgatccttagtctggcct			
q02726fw	*PDK*	acctcccgttcagctagtct	this study	138	94
q02726rv		aattcatcagagtcggcccc			
RibPro 3 f	*RPS9*	gtgaatgggagaccaatctcag	this study	128	94
RibPro 3 r		ttgcctcctccatgagtcag			
q00682fw	*RPS11*	ggactgggcttcaagactcc	this study	97	86
q00682rv		gaatcctgcccctgatcgag			
q00153fw	*RPS14*	agttgctcgagtgactggtg	this study	86	89
q00153rv		catcttgggcagccaacatg			
q00241fw	*SDH*	caatcgcctgaggaccgtaa	this study	80	88
q00241rv		ctggggcaacttgtagagca			
Ubc 1 f	*Ubc*	cggaccagtacccttacaaatc	this study	128	93
Ubc 1 r		atcaaacatcggcgaccag			
UbcE2 3 f	*UbcE2*	gtcgaactgtgacgagtttg	this study	117	89
UbcE2 3 r		acggccttagtcttcgatg			

That *C*q values for the *in planta* samples are higher than the *C*q values for the *in vitro* samples, especially the germinated spore, is mostly due to the fact, that here fungal RNA is mixed with plant RNA. At the later time points, *C*q values in the plant samples decrease; this tendency reflects the increase of fungal biomass in infected leaves.

Since spores are resting structures with no metabolic activity, it is not surprising that transcript levels of most genes are relatively low in this sample. Nevertheless, the fact that there are large differences in transcript levels between spores and germ tubes, also for housekeeping genes, is a highly relevant finding, especially in light of the fact that many studies on gene expression in fungi give expression levels relative to those in spores [20]. What was surprising to us, are the generally high *C*q values for the appressorium sample. There is no fundamental reason why expression levels should be lower in appressoria than in germ tubes. One explanation could be the way the appressorium structures are prepared. As described in Section 3.1.2 these structures are grown on polyethylene (PE) sheets for a relatively long time. After 16 h it must be expected that, while some specimens are still growing and forming appressoria, many others are no longer viable, and the RNA within is already degrading. At this stage also contaminations with bacteria or other fungi may be higher than in the germinated spore stage. This in turn would lead to higher *C*q values despite the fact that RNA was quantified and tested on a gel—and considered good quality.

Judging from the ranges of *C*q values, *CytB* and *SDH* are the most stable genes in the *in vitro* samples (Figure 1b). However, even the range for *CytB* with 6.5 is still high. For the *in planta* samples the ranges are generally lower and would be lower still if not for the changes caused by the increase of fungal biomass in the leaf. Here, *RPS11* and *RPS14* can be rated as most stable (Figure 1c). Over all samples (Figure 1d), again *CytB* has the smallest range (8.0), followed by *Act* and *RPS14*.

In analyses with the algorithms geNorm and NormFinder other genes are considered the most stable (Figure 2). geNorm analysis ranks best *RPS14* and *UbcE2* for *in vitro* samples, *PDK* and *CytB* for *in planta* samples, and *PDK* and *Ubc* for all samples. Results of the NormFinder analysis are relatively similar to those of the geNorm analysis. With NormFinder *RPS14* and *UbcE2* are ranked best for *in vitro* samples, *GAPDH* and *CytB* for *in planta* samples, and *PDK* and *RPS14* for all samples.

However, there are big differences how the genes are ranked between the different combinations of samples. While *PDK* and *RPS14* that are ranked best for all samples by NormFinder are also good in the *in vitro* and *in planta* subsets of samples, *Ubc*, ranked second for all samples by geNorm, is ranked much lower, especially in the *in planta* subset. Most striking, however, is the difference in ranking for *CytB* that is ranked among the best for the *in planta* samples, but worst for the *in vitro* samples and for the combination of all samples.

The rankings, especially for *CytB*, are also contrary to what could be deduced from the *C*q ranges. While *CytB* is overall the most stable gene based on its *C*q range, it is ranked worst by both algorithms for all samples and for the *in vitro* samples. Since both algorithms are based on comparing the difference between the *C*q values in different samples, they will rank genes that have a similar regulation better than genes that have constant *C*q values but differ in their regulation from other genes. This is obviously the case for *CytB*. *CytB* is highly expressed but its expression in the germ tube is not as much elevated as that of most of the other genes (Figure 1a). So it seems that the higher stability of *CytB* is responsible for its lower ranking by geNorm and NormFinder.

It is hard to give a recommendation for what genes should actually be used for normalization for *in vitro* samples and for all samples. On the one hand *CytB*, *SDH*, and *Act* are the most stable genes, while on the other hand *RPS14* and *UbcE2* go best with the tendency of all other genes. Therefore, normalizing with either *CytB* or *RPS14* and *UbcE2* could be recommended. *CytB* would be particularly useful, if one would like to get close to absolute quantification with normalization. Using *RPS14* and *UbcE2* would be more suitable if researchers have the intention to mask the elevated metabolic activity in the germ tube.

The results for the *in planta* samples are better and much more straightforward. Here we recommend use of *CytB* in combination with either *PDK* or *GAPDH* for normalization.

Expression of potential reference genes is obviously not stable between the three *in vitro* samples tested; choosing the right reference gene(s) is difficult, and consequently analysis of gene expression during the *in vitro* stages will be precarious. Following the arguments detailed above the fault lies with the spore and the appressorium samples. For reliable gene expression analyses one should therefore consider to omit these samples. We strongly recommend not to use spores as the sample to which expression in other samples should be compared to, but rather use the germ tube stage as the reference sample. When spore and appressorium are included in expression studies we recommend that expression changes between the *in vitro* stages should only be considered relevant if they are greater than a thousand fold, since even for the most stable gene tested expression varies roughly a hundredfold.

To identify the most suitable gene(s) for normalizing in a sample set omitting spore and appressorium stages another analysis was performed. This combines *in planta* samples with germ tube as the only *in vitro* sample (Figure 2g,h). Here *Elf3* and *RPS9* or *PKD* and *GAPDH* were identified as the most suitable reference genes. Consequently, we recommend to include only *in planta* stages, and a germ tube sample, and to use a combination of the above genes for normalization in studies that do not necessarily need to include resting urediospores and appressorium stages.

### 2.4. Reference Genes for G. max: Ukn2 and cons7 Can Be Used for Normalizing RT-qPCR in Tissues Infected with Soybean Rust and Also for Superinfection of Soybean Rust on BPMV

During determination of primer efficiencies (Table 4) the primer pairs chosen for *CYP2* (Gm CYP2 f and Gm CYP2 r) and *ELF1B* (Gm ELF1B f and Gm ELF1B r) gave rise to unspecific side products; consequently, these genes were excluded from further analyses.

**Figure 3:**
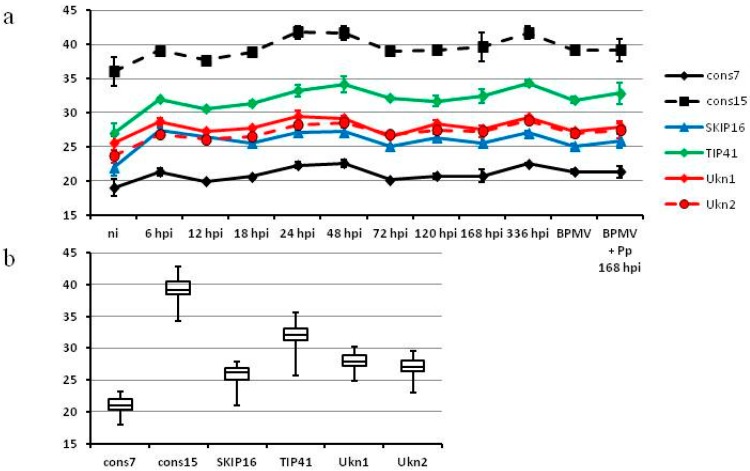
Variation of *C*q values for *G. max* candidate reference genes between samples (vertical axis: *C*q). (**a**) Average and standard deviation of *C*q values for every sample; (**b**) box plots representing the range of *C*q values; For better representation of RNA abundance the *C*q values were corrected for primer efficiency: *C*q_corr_ = lg_2_ ((2 × e)*^C^*^q^).

After RT-qPCR for the other genes and all samples, the ranges of corrected *C*q values suggested *cons7*, *Ukn1*, and *Ukn2* as the most stable genes (Figure 3). Even though the genes differ strongly in their expression, all appear relatively stable over the tested samples.

**Figure 4:**
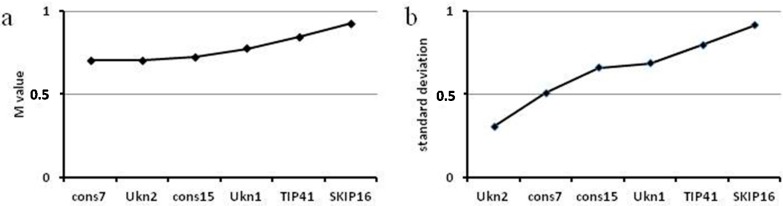
Ranking of *G. max* candidate reference genes for stability by the algorithms geNorm (**a**) and NormFinder (**b**).

Ranking of the genes by geNorm and NormFinder identified *Ukn2* and *cons7* as the most stable genes (Figure 4). In this case we find no discrepancies between our *C*q range analysis and results of the algorithms. We therefore recommend using *cons7* and *Ukn2* for normalization in expression profiling of soybean genes during soybean rust infection and for quantification of either virus or fungus during HbV experiments.

More studies dealing with reference genes were only found, or were only published, after we finished our experiments [29,30,31,32]. In addition to genes selected from [24] these studies include other frequently used reference genes. Of special interest here is [30] who included infection with Soybean Mosaic Virus in their study and found *ELF1B* and *Ukn2* to be most stable under these conditions. Closest to our own study is [32] which included BPMV infection and here found an ABC transporter and elongation factor 1 α most stably expressed. During infection with powdery mildew, another biotrophic fungus, they found *CYP2* and tubulin α-4 most stable. So while this goes well together with our result for *Ukn2*, this also suggests that there are alternatives to our findings. Also it seems most regrettable in retrospect that *CYP2* and *ELF1B* did not work in our hands.

### 2.5. Number of Reference Genes Needed for Efficient Normalization

Since generally no single gene can be considered truly and absolutely stable, it is also accepted practice to use more than one gene for normalization, expecting that expression changes in the different genes will balance each other. Besides determining the most stable gene, the NormFinder algorithm also calculates a value designated accumulated standard deviation. This value indicates the optimal number of genes to be used for normalization.

**Figure 5:**
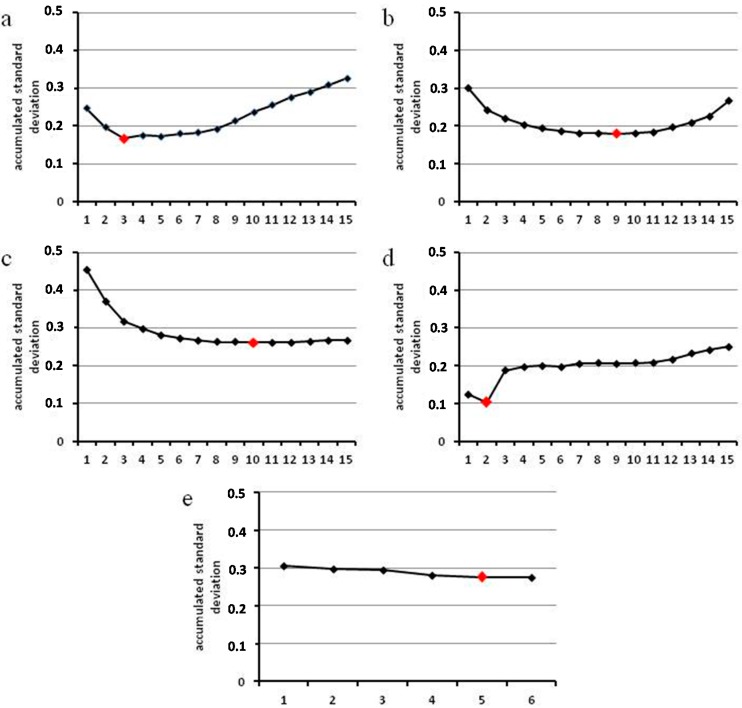
Number of reference genes recommended by NormFinder (optimal number marked red); (**a**) for *P. pachyrhizi in vitro* structures; (**b**) for *P. pachyrhizi in planta* structures; (**c**) for all *P. pachyrhizi* samples; (**d**) for all *P. pachyrhizi* samples excepting spore and appressorium; (**e**) for *G. max*.

Figure 5 shows our results for these analyses in the different sample sets. The optimal numbers differ strongly for different sample sets or combinations of samples. In two cases we have a steep drop of the accumulated standard deviation up to the optimal gene number, in two cases the drop flattens out and the optimal number is reached much later. For the plant genes there is altogether only little alteration between different numbers of genes.

We observe that there are bigger changes between the first few genes whereas differences for higher numbers are less important. We therefore ignore the optimal numbers for *in planta* samples and all samples (Figure 5b,c), and assume that a lesser number of genes for normalization is also adequate. Of course this is also a question of cost and effort, and here using nine, ten, or five genes, respectively, for normalization is too much. Since in this case the plant genes all have good stability, using fewer genes is justified, and we therefore recommend lower numbers.

Above we are recommending gene combinations for normalization; these combinations always include what we consider the right number of genes.

Overall the gene combinations we recommend provide a good option for normalization in the studied sample combinations if researchers heed the mentioned caveats, especially limited stability in the *in vitro* structures. Studies by Vieira *et al.* [19] and Hacquard *et al.* [20] suggest using DNA for additional normalization or as a correction for the normalization with reference genes. Even though our results for the *in vitro* structures seem to suggest that additional normalization is necessary, using DNA for these structures might not be appropriate, since during appressorium formation also nuclear division takes place, so the DNA content increases even though up to this point the biomass stays the same. If researchers need additional normalization we would rather recommend to measure the amount of total RNA used in reverse transcription and take this into account. Only for direct comparisons between resting spore and germ tube, where DNA content stays constant, DNA could be used for normalization. In this case however we would recommend using DNA only, without combining it with RNA references. On the other hand for the *in planta* structures we find that the tested genes are stable and are reflecting the increase in biomass, so for these samples additional normalization is clearly not necessary.

For the soybean genes, we found that all those tested have good stability, which could be expected, judging from the amount of samples that were already tested in the studies the candidates were chosen from [22,23,24]. Nevertheless, our study now provides evidence that these genes can be used to track expression changes during rust infection and in host-induced gene silencing.

## 3. Experimental Section

### 3.1. Plant Material, Fungal Isolate, Inoculation with Virus and Rust Fungus

#### 3.1.1. Plant Material

Soybean (*G. max*) cultivar Thorne (Bayer CropScience AG, Lyon, France) was grown in the greenhouse with 16 h light/8 h dark at 22 °C. Plants were transplanted after formation of the primary leaves.

#### 3.1.2. Fungal Isolate, Plant Inoculation and *in Vitro* Structures

Urediospores of isolate Thai 1 (laboratory collection, Phytopathology, University of Hohenheim, Stuttgart, Germany) were first hydrated by stirring vigorously for 45 min in a watery suspension with 0.2% urediospores, 0.2% milk powder, 0.01% Tween20. This suspension was then sprayed on 21 days old *G. max* plants (in case of superinfection of *P. pachyrhizi* on BPMV, plants 21 days after virus inoculation (see below)) using a chromatography vaporizer. Plants were then kept in the dark for 12 h at 95% humidity and 20 °C. After that plants were returned to the greenhouse until sampling or for collection of urediospores that appeared starting at 9 dpi.

Germ tubes (gt) were obtained as described by [3] by spreading urediospores on the water surface and incubation for 12 h at room temperature in the dark.

To induce *in vitro* formation of appressoria (ap), the same suspension used for plant inoculation was sprayed on PE sheets instead and incubated for 16 h at 95% humidity at room temperature in the dark.

#### 3.1.3. BPMV Inoculation

Plasmids pBPMV-IA-R1M and pBPMV-IA-V1 [33] were introduced into soybean plants using particle bombardment as described [34]. 21 dpi symptomatic leaves were harvested, dried and frozen. This infected plant material was homogenized using mortar and pestle, mixed with Sörensen inoculation buffer (1 M NaH_2_PO_4_/Na_2_HPO_4_, pH 7.2), and with the help of carborundum the mix was rubbed onto freshly developed primary leaves of soybean plants. The carborundum was washed off again and plants were kept in the dark at 95% humidity for 12 to 16 h before they were returned to the greenhouse.

### 3.2. Sample Homogenization and RNA Isolation

Homogenization was performed using the FastPrep-24 (MP Biochemicals GmbH, Eschwege, Germany) for 20 s at 4.5 m/s.

RNA isolation was performed using the Plant RNA Isolation Mini Kit (Agilent Technologies, Santa Clara, CA, USA). We followed the procedure described in the kit protocol except for adding an additional centrifugation step (1 min, 16,000× *g*, room temperature) between homogenization and addition of the material to the prefiltration column.

Below are listed particulars for the different sample materials:

Fifty mg urediospores were used directly from storage at −80 °C. Together with two metal beads they were put in a homogenization tube, cooled in liquid nitrogen and homogenized deep frozen. Afterwards 600 μL Extraction Solution was added.

Germ tubes were removed from the water surface by filtering. Together with 600 μL Extraction Solution roughly 100 mg germ tubes were added to a homogenization tube with Lysing Matrix E.

Six hundreds μL Extraction Solution were dispersed on the PE membrane with appressorial structures and the structures scraped together with a rubber and transferred to a homogenization tube with Lysing Matrix E.

One hundred mg of leaf material were excised using a cork borer. Together with two metal beads they were put in a homogenization tube, cooled in liquid nitrogen and homogenized deep frozen. Afterwards 600 μL Extraction Solution were added.

Integrity of the RNA and absence of contaminations were checked by gel electrophoresis using 1% agarose gels with TAE buffer containing 1% bleach (6% NaClO). RNA concentrations were measured using a Qubit^®^ 2.0 fluorometer (Life technologies, Darmstadt, Germany) with the Qubit^®^ Broad Range Assay Kit (Life technologies). Storage of the RNA was as precipitate in 0.3 M sodium acetate (pH 5.3) and 70% ethanol at −80 °C.

### 3.3. RT-qPCR

#### 3.3.1. Reverse Transcription

Before reverse transcription, DNA contaminations were removed by digestion with PerfeCTa^®^ DNase I (Quanta Biosciences, Gaithersburg, MD, USA) using 5 μg total RNA in a 10 μL reaction.

For reverse transcription the Tetro cDNA Synthesis Kit (Bioline Reagents Ltd., London, UK) was used. The 10 μL from DNase I digestion were directly used in this reaction; we followed the recommendations of the manufacturer using random hexamer primers. cDNA was stored at −80 °C.

#### 3.3.2. Machinery, Chemistry, and Reaction Conditions of Real Time PCR

Real Time PCR reactions were run on a CFX96™ Real-Time-PCR System (Bio-Rad Laboratories, Hercules, CA, USA).

We were using the SensiFAST™ No-ROX mix (Bioline Reagents Ltd., London, UK). Primers were used at a concentration of 0.3 μM; we used 2 μL of cDNA in 20 μL reactions. PCR cycling followed a two step protocol with an initial denaturation step of 95 °C for 5 min, and then 5 s 95 °C and 15 s at 60 °C. 39 cycles were completed; then a melt curve analysis was run: dissociation at 95 °C for 10 s, then the actual melt curve spanning 65 to 95 °C with 0.5 °C temperature increase per 5 s.

#### 3.3.3. Data Analysis

Data logging and the determination of *C*q, Δ*C*q, and ΔΔ*C*q values was done using Bio-Rad CFX Manager 2.1 (Bio-Rad Laboratories). We used automatic threshold determination.

As proposed by [25] we considered primer efficiency in our analysis. Primer efficiencies were determined using rows of four 1:10 dilutions of cDNA derived from germ tubes and 7 dpi infected plant material for *P. pachyrhizi* primers and also 7 dpi plant material for *G. max* primers. The analysis was performed in biological triplicate and technical replicate.

Both for determination of primer efficiency and determination of the most stable candidate reference genes, we used the GenEx software package: GenEx 6.0.1.612 (MultiD Analyses AB, Göteborg, Sweden).

### 3.4. Primers

Many sequences for primers used in this study were taken from previous studies (see references in Table 3 and Table 4). Otherwise we used the webtool Primer3 [35,36] for primer design. We set the following parameters: primer size: minimum 18 bases, optimum 20 bases, maximum 25 bases, primer Tm: min 58 °C, opt 60 °C, max 62 °C; primer GC%: min 30, opt 50, max 70. The product size range was set to 75–150 bp.

The products of the PCR reactions for determination of primer efficiency were also loaded on TAE (tris, acetate, ethylenediaminetetraacetic acid) agarose gels and only when a clear single band was obtained the genes were analyzed further.

**Table 4:** Primers used in qRT-PCR for *G. max* genes.

Name	Gene	Sequence	Reference	Amplicon [bp]	Efficiency [%]
Gm cons7 f	*cons7*	atgaatgacggttcccatgta	[23]	114	81
Gm cons7 r		ggcattaaggcagctcactct			
Gm cons15 f	*cons15*	taaagagcaccatgcctatcc	[23]	97	106
Gm cons15 r		tggttatgtgagcagatgcaa			
Gm CYP2 f	*CYP2*	cgggaccagtgtgcttcttca	[22]	154	na
Gm CYP2 r		cccctccactacaaaggctcg			
Gm ELF1B f	*ELF1B*	gttgaaaagccaggggaca	[22]	118	na
Gm ELF1B r		tcttaccccttgagcgtgg			
Gm SKIP16 f	*SKIP16*	gagcccaagacattgcgagag	[24]	60	87
Gm SKIP16 r		cggaagcggagaactgaacc			
Gm TIP41 f	*Tip41*	aggatgaactcgctgataatgg	[24]	88	95
Gm TIP41 r		cagaaacgcaacagaagaaacc			
Gm UKN1 f	*Ukn1*	tggtgctgccgctatttactg	[24]	74	90
Gm UKN1 r		ggtggaaggaactgctaacaatc			
Gm UKN2 f	*Ukn2*	gcctctggatacctgctcaag	[24]	79	96
Gm UKN2 r		acctcctcctcaaactcctctg			

## 4. Conclusions

Our results show that there are enormous changes in gene expression even among housekeeping genes during early development of soybean rust. There is only little gene expression in resting spores and there may be problems with viability of *in vitro* structures in the appressorium stage. We therefore recommend to specify gene expression data relative to the germ tube stage rather than the resting urediospore stage. We also suggest that gene expression changes in the appressorium should only be considered relevant when they are a thousand fold or higher. We could identify reference genes that are suitable for expression profiling during the infection process *in planta* and combined with the germ tube stage: *CytB*/*PDK* or *CytB*/*GAPDH* and *Elf3*/*RPS9* or *PKD*/*GAPDH* are suitable reference gene combinations.

We could show that reference genes *Ukn2* and *cons7* are most stable in our experimental setup and are best suited for normalization of expression of soybean genes during soybean rust infection and in HbV experiments.
